# Comparative genome and phylogenetic analysis revealed the complex mitochondrial genome and phylogenetic position of *Conopomorpha sinensis* Bradley

**DOI:** 10.1038/s41598-023-30570-7

**Published:** 2023-03-27

**Authors:** Hong Chang, Jianglong Guo, Mingzhi Li, Yan Gao, Siwei Wang, Xiaonan Wang, Yanping Liu

**Affiliations:** 1 Institute of Plant Protection, Guangdong Academy of Agricultural Sciences, Key Laboratory of Green Prevention and Control on Fruits and Vegetables in South China Ministry of Agriculture and Rural Affairs, Guangdong Provincial Key Laboratory of High Technology for Plant Protection, Guangzhou, 510640 China; 2 Key Laboratory of Integrated Pest Management On Crops in Northern Region of North China, Ministry of Agriculture and Rural Affairs, IPM Center of Hebei Province, Plant Protection Institute, Hebei Academy of Agricultural and Forestry Sciences, Baoding, 071000 China; 3 Bio&Data Biotechnologies Co. Ltd., Guangzhou, 510640 China

**Keywords:** Comparative genomics, Phylogenetics

## Abstract

*Conopomorpha sinensis* Bradley is a destructive pest that causes severe economic damage to litchi and longan. Previous *C. sinensis* research has focused on population life tables, oviposition selectivity, pest population prediction, and control technology. However, there are few studies on its mitogenome and phylogenetic evolution. In this study, we sequenced the whole mitogenome of *C. sinensis* by the third-generation sequencing, and analyzed the characteristics of its mitogenome by comparative genome. The complete mitogenome of *C. sinensis* is a typical circular and double-stranded structure. The ENC-plot analyses revealed that natural selection could affect the information of codon bias of the protein-coding genes in the mitogenome of *C. sinensis* in the evolutionary process. Compared with 12 other Tineoidea species, the *trnA*-*trnF* gene cluster of tRNA in the *C. sinensis* mitogenome appears to have a new arrangement pattern. This new arrangement has not been found in other Tineoidea or other Lepidoptera, which needs further exploration. Meanwhile, a long AT repeated sequence was inserted between *trnR* and *trnA*, *trnE* and *trnF*, *ND1* and *trnS* in the mitogenome of *C. sinensis*, and the reason for this sequence remains to be further studied. Furthermore, the results of phylogenetic analysis showed that the litchi fruit borer belonged to Gracillariidae, and Gracillariidae was monophyletic. The results will contribute to an improved understanding of the complex mitogenome and phylogeny of *C. sinensis*. It also will provide a molecular basis for further research on the genetic diversity and population differentiation of *C. sinensis*.

## Introduction

*Conopomorpha sinensis* Bradley (Gracilariidae, Lepidoptera) is the most important borer affecting the production of litchi and longan. It is mainly distributed in the litchi-producing areas of China, India, Nepal, and Southeast Asia. *C. sinensis* larvae eat the flower ears, leaves, shoots, and fruits of litchi and longan. The larvae are especially damaging to the fruits. The fruit decay rate ranges from 10 to 20%, and sometimes can reach 90%. It significantly reduces the fruit quality, reduces economic value and causes great economic losses to the litchi and longan industry^1,2^. *C. sinensis* research mainly focuses on biology, physiology and biochemistry, pest population prediction, and control technology. There are few studies on the insect source relationship and population genetics within the main distribution of *C. sinensis*^2–5^. Based on the transcriptome data of *C. sinensis*, the SSR locus information of *C. sinensis* was analyzed. The results provide a basis for studying the population genetic diversity of litchi fruit borer^6^.

Mitochondrial DNA (mtDNA) is commonly used as a molecular marker in population evolutionary and genetics biology due to its simple technical procedures, conservation in various organisms, and maternal inheritance. The organization and structure of mtDNA are simple and highly conserved, and mtDNA has a more rapid rate of evolutionary change than nuclear DNA^7–9^. The mitogenomes of insects are typically circular, double-stranded molecules with a length between 15 and 18 kb^10^. A total of 37 genes are translated, including 13 protein-coding genes (PCGs), 22 transport RNA (tRNA), and 2 ribosomal RNA (rRNA) genes^11,12^. There is also a noncoding region of variable size, termed the A + T-rich region or the control region. The control region regulates and initiates mitochondrial genes replication and transcription^13^. In addition, the length of the control area varies, and differences in mitochondrial genome length are mainly caused by changes in the A + T rich region length^14^.

The mitochondrial genomes of many insect species have been obtained, and mitochondrial genomes have been used in phylogenetics and population genetics studies of many insects^8,10,15,16^. Mitochondrial genomes have been used to study the genetic structure and phylogeography of locust populations^17^. The evolutionary history of Neuropterida was illuminated by mitochondrial phylogenomics^18^. The phylogeography of 20 populations of *Neoneuromus ignobilis* was studied using mitochondrial genomic and microsatellite datasets^19^. The biogeographical history of Megaloptera was studied using mitochondrial genome sequences^20^.

The mitochondrial genome of *C. sinensis* was sequenced and spliced by high-throughput sequencing technology and its basic characteristics were analyzed in this study. We also used comparative genomics to analyze the mitochondrial genomes of 13 Tineoidea species including *C. sinensis*. The nucleic acid composition, base preference, and gene arrangement were compared to clarify the characteristics of the mitogenome of litchi fruit borer. In addition, we constructed a phylogenetic tree based on the mitochondrial whole genome sequence to study the phylogenetic position of litchi fruit borer in the Tineoidea. The results will contribute to an improved understanding of the complex mitochondrial genome and phylogeny of *C. sinensis*. It also will provide a molecular basis for further research on the genetic diversity and population differentiation of *C. sinensis*.

## Results

### The whole mitogenome composition of *C. sinensis*

**Figure 1:**
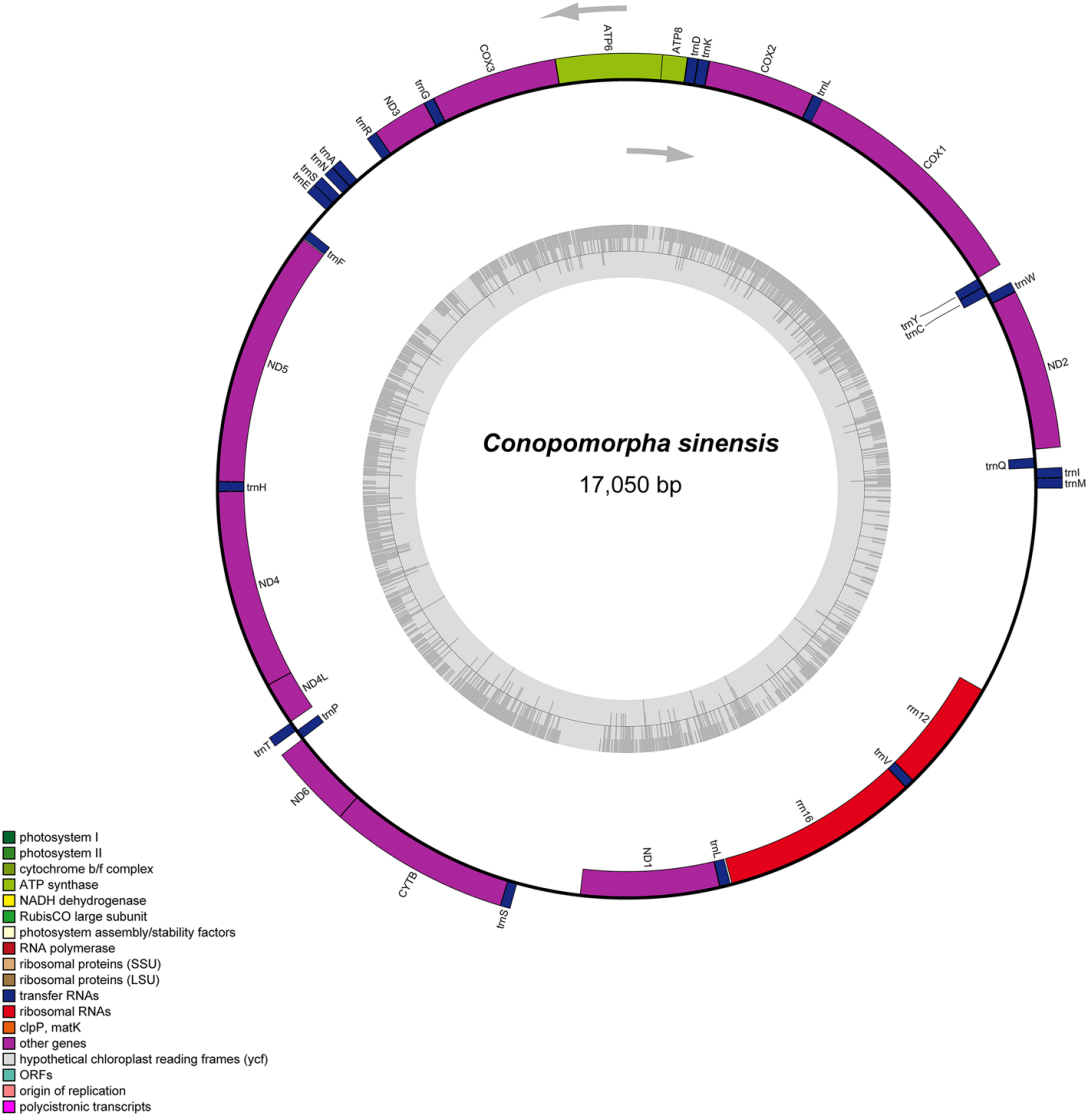
Mitochondrial genome map of *C. sinensis*. Colors in the figure represent different types of regions and genes. Detailed information is shown in the figure column.

The mitogenome of *C. sinensis* (GenBank accession number OK310517) is a typical circular double-stranded structure with a length of 17,050 bp (Fig. 1). The mitogenome of *C. sinensis* contains 37 typical genes, including 13 protein-coding genes (PCGs), 22 transport RNA (tRNA), 2 ribosomal RNA (rRNA) genes, and a 1390 bp A + T-rich area. Among them, the majority strand (J-strand) contains 14 tRNAs and 9 PCGs genes, and the minority strand (N-strand) contains 8 tRNAs, 2 rRNAs, and 4 PCGs genes.

In the whole mitogenome of *C. sinensis*, the nucleotide composition is as shown in Table 1. The content of A + T (83.52%) was significantly higher than that of C + G (16.48%) in the whole mitogenome. The overall G + C content of the mitogenomes ranged from 16.48 to 20.14% among other Tineoidea insects^21–23^ (Supplementary Table S1). Similarly, A + T content was higher than G + C content in the PCGs, tRNAs, and rRNAs. The analysis of the complete mitogenome nucleotide skew shows that it has a negative GC-skew value (− 0.17), while a positive AT-skew value (0.01).

**Table 1:** Base composition of the mitogenomes of *C. sinensis*.

Regions	Length (bp)	A (%)	T (%)	C (%)	G (%)	AT (%)	CG (%)	AT Skew	GC Skew
Mitogenomes	17,050	42.25	41.27	9.62	6.86	83.52	16.48	0.01	− 0.17
PCGs	11,205	34.70	45.78	9.19	10.33	80.48	19.52	− 0.14	0.06
tRNAs	1472	42.66	39.88	7.27	10.19	82.54	17.46	0.03	0.17
rRNAs	2104	43.11	42.59	4.75	9.55	85.69	14.31	0.01	0.34
D-loop	1389	45.36	49.89	3.24	1.51	95.25	4.75	− 0.05	− 0.36

### PCGs characterization of *C. sinensis*

The size of the 13 protein-coding genes of *C. sinensis* is 11,205 bp, which accounts for 65.72% of the whole mitogenomes (Table 1). Similar to the whole genome, A + T content (80.48%) in PCGs was higher than G + C content (19.52%). The AT-skew value in PCGs genes is negative (− 0.14), while the GC-skew value is positive (0.06), reflecting the preference for T and G bases compared to A and C bases (Table 1).

All of the initiation codons of PCGs were the ATT or ATG codons, except *COI* gene, which started with CGA, and all of the PCGs were terminated with TAN or truncated T residue. The RSCU values of *C. sinensis* are shown in Table 2 and Supplementary Fig. S1. Among the 20 amino acids (Table 2), 17 amino acids are translated by 2 condons, 3 condons, 4 codons or 6 codons, except Aspartic (Asp), Cysteine (Cys) and Glutamine (Gln). Among them, the RSCU value of 29 codons is greater than 1 (1.26–5.46), indicated that these codons were used more frequently. At the same time, the results showed that all high frequency codons (RSCU > 1) used A or U base as the third codon. The most commonly used codons are GCU-Ala, CGA-Arg, GGA-Gly, AUU-Ile, UUA-Leu, AUA-Met, CCU-Pro, UCU-Ser, ACU-Thr, and GUA-Val, while GAC-Asp, CAG-Gln, CUC-Leu, CUG-Leu, CCG-Pro, UGC-Cys, AGC-Ser, AGG-Ser, and ACG-Thr are not present in the PCGs (Supplementary Fig. S1).

**Table 2:** Frequency and RSCU values of codon in PCGs in the mitogenome of litchi fruit borer.

Amino acid	Codon	Count	RSCU	Amino acid	Codon	Count	RSCU	Amino acid	Codon	Count	RSCU
Ala	GCU	60	2.14	His	CAU	61	1.85	Ser	UCU	113	2.78
	GCA	45	1.61		CAC	5	0.15		AGA	88	2.17
	GCC	5	0.18	Ile	AUU	433	1.95		UCA	83	2.04
	GCG	2	0.07		AUC	12	0.05		AGU	30	0.74
Arg	CGA	32	2.46	Leu	UUA	492	5.46		UCC	8	0.20
	CGU	17	1.31		CUA	27	0.30		UCG	3	0.07
	CGG	2	0.15		CUU	12	0.13		AGC	0	0.00
	CGC	1	0.08		UUG	10	0.11		AGG	0	0.00
Asn	AAU	235	1.87		CUC	0	0.00	Ter	UAA	11	1.69
	AAC	17	0.13		CUG	0	0.00		UAG	2	0.31
Asp	GAU	62	2.00	Lys	AAA	96	1.79	Thr	ACU	86	2.28
	GAC	0	0.00		AAG	11	0.21		ACA	62	1.64
Cys	UGU	28	2.00	Met	AUA	279	3.61		ACC	3	0.08
	UGC	0	0.00		AUG	25	0.32		ACG	0	0.00
Gln	CAA	60	2.00		AUU	4	0.05	Trp	UGA	90	1.94
	CAG	0	0.00		CGA	1	0.01		UGG	3	0.06
Glu	GAA	65	1.76	Phe	UUU	355	1.86	Tyr	UAU	207	1.88
	GAG	9	0.24		CCU	65	2.13		UAC	13	0.12
Gly	GGA	114	2.28	Pro	CCA	52	1.70	Val	GUA	62	2.00
	GGU	63	1.26		CCC	5	0.16		GUU	56	1.81
	GGG	21	0.42		UUC	26	0.14		GUG	5	0.16
	GGC	2	0.04		CCG	0	0.00		GUC	1	0.03

### RNA characterization of *C. sinensis*

**Figure 2:**
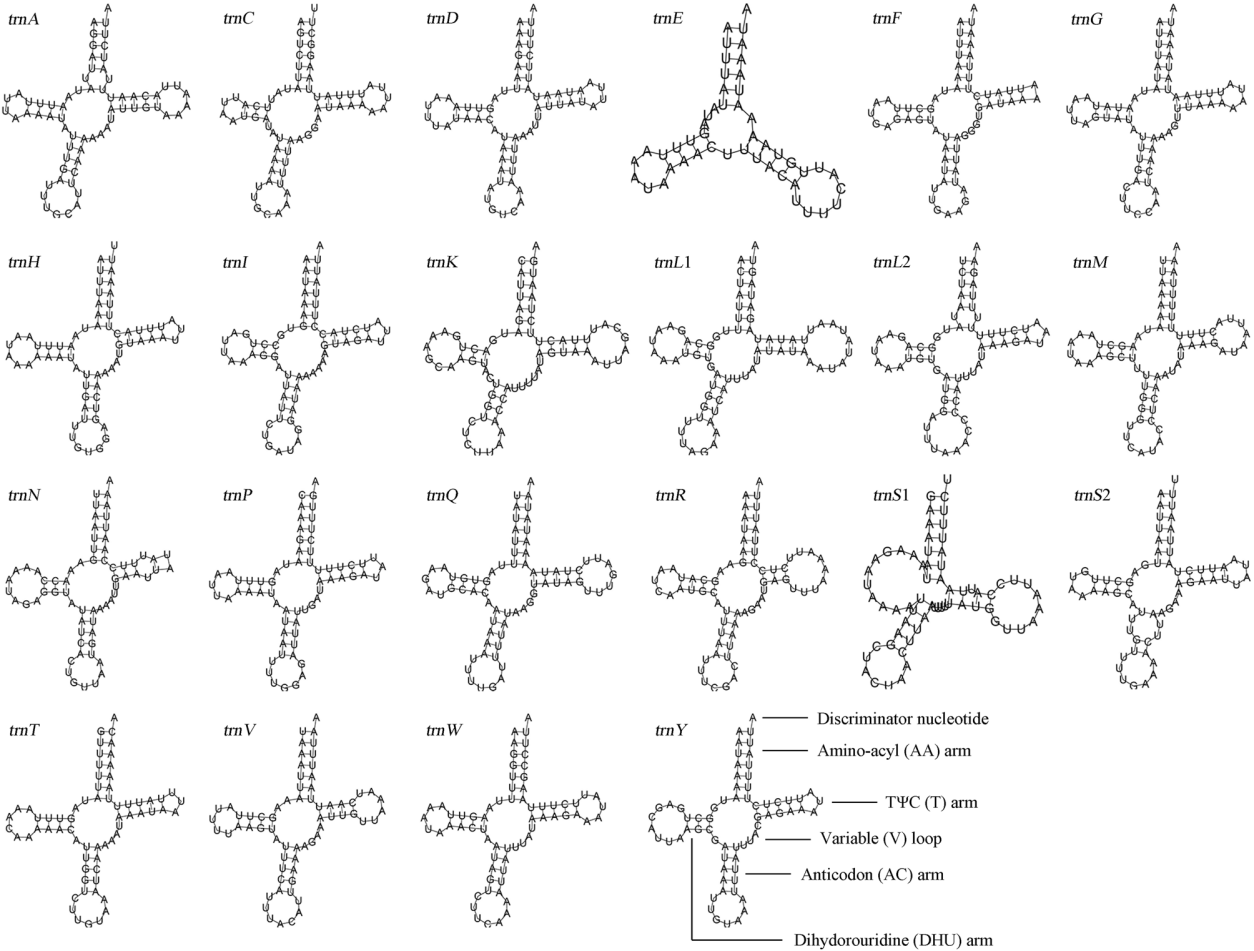
The secondary structure of tRNAs predicted by MITOS2 software in *C. sinensis* mitogenomes.

The 22 typical transport RNA genes were all discovered in the mitogenome of *C. sinensis* (Fig. 1). The entire length of the 22 tRNA was 1472 bp and ranged from 64 bp (*trnF*) to 74 bp (*trnL*) in length. Both the values of GC-skew (0.17) and AT-skew (0.03) in tRNA genes are positive, showing the preference for A and G bases compared to T and C (Table 2). Most of the tRNAs are classic clover-leaf structures, except *trnE* without the pseudoricinr (TΨC) stem and *trnS1* lacking the dihydrouridine (DHU) stem (Fig. 2).

The 12S rRNA (*rrnS*, 763 bp) and 16S rRNA (*rrnL*, 1335 bp) were at conserved positions on the N-strand of the mitogenome of litchi fruit borer, which were located on the *trnV* and the control region, and between *trnL* and *trnV*, respectively (Fig. 1). Similar to the tRNAs, the AT-skew (0.03) value and GC-skew (0.17) are positive for the rRNAs, which indicates a slight preference for A and G bases compared to T and C bases (Table 1).

### Control region characterization of *C. sinensis*

The control region is situated between the *rrn12* and *trnM* genes, with a size of 1,389 bp (Fig. 1, Table 1). The content of A + T (95.25%) was significantly higher than that of CG (4.75%). The value of AT-skew (− 0.05) and GC-skew (− 0.36) in the control region are negative, reflecting the preference for T and C bases compared to A and G bases. The control region appears to be the promoter of mitochondrial genome transcription and replication^24^.

### Characteristic analysis of comparative genome

The mitochondrial genome of *C. sinensis* was used as a reference and comparative genome analysis of species from different families in the Tineoidea was conducted to analyze mitochondrial genome composition and construct the structural variation map. The nucleotide composition and characteristic analysis of mitochondrial genomes of 13 Tineoidea species are shown in Supplementary Table S1. The complete mitogenomes of Tineoidea used in this study ranged from 15,027 bp (*Amorophaga japonica*) to 17,050 bp (*C. sinensis*) in length. The length of the control region changes greatly compared with other genes in mitochondria in the Tineoidea species (Supplementary Table S1). These results demonstrated that the length of the mitochondrial genome was mostly determined by the size of the control region among Tineoidea species, which is similar to other insects^25,26^.

The mitochondrial genome of *C. sinensis* contains 42.25% A base, 41.27% T base, 9.62% C base, and 6.86% G base (Table 1). Compared to other Tineoidea insects, the mitochondrial genome of litchi fruit borer has a higher A + T content (83.52%). The A + T content of Tineoidea moths ranges from 79.86% (*Eudarcia gwangneungensis*) to 83.52% (*C. sinensis*), with an A + T average of 81.36% (Supplementary Table S1). Comparing the A + T content of the complete mitogenome, the control region, , tRNAs, rRNAs,,and PCGs, the A + T content in the control region was the highest, while the A + T content in PCGs genes was the lowest for the 13 Tineoidea species (Supplementary Table S1). The GC skew of the full mitogenome within the 13 species varied from − 0.30 to − 0.14; however, the AT skew varied from − 0.03 to 0.05. These results show that, compared with other Tineoidea insects, the whole mitogenome of litchi fruit borer has a higher AT skew value and the lowest CG skew. Previous studies have shown that base composition bias is related to mitochondrial genome replication and transcription^27,28^.

We calculated the ENC values to analyze the base bias of the PCGs of 13 Tineoidea species. The ENC value varies greatly among different species, from 16.59 to 39.96, indicating that the codon bias of different species is different (Supplementary Table S2). Among the 169 protein-coding genes, only 17 genes had ENC values higher than 35, accounting for only 10.06% of the total genes, indicating that most protein-coding genes have a high codon usage bias. Furthermore, the results showed that the ENC values of the six protein genes (ATP6, ND1, ND3, ND4, ND5 and ND6) among 13 Tineoidea species were less than 35, indicating that these protein-coding genes have high codon preference. In addition, the ENC value of the same gene varied greatly in different species. For example, the ENC value of *ATP8* gene was 16.59 in *Amorophaga japonica* and 39.96 in *Clania variegata*. The results indicated that for the same protein-coding gene, some species had strong codon preference, while others had low preference (Supplementary Fig. S2 and Table 2).

**Figure 3:**
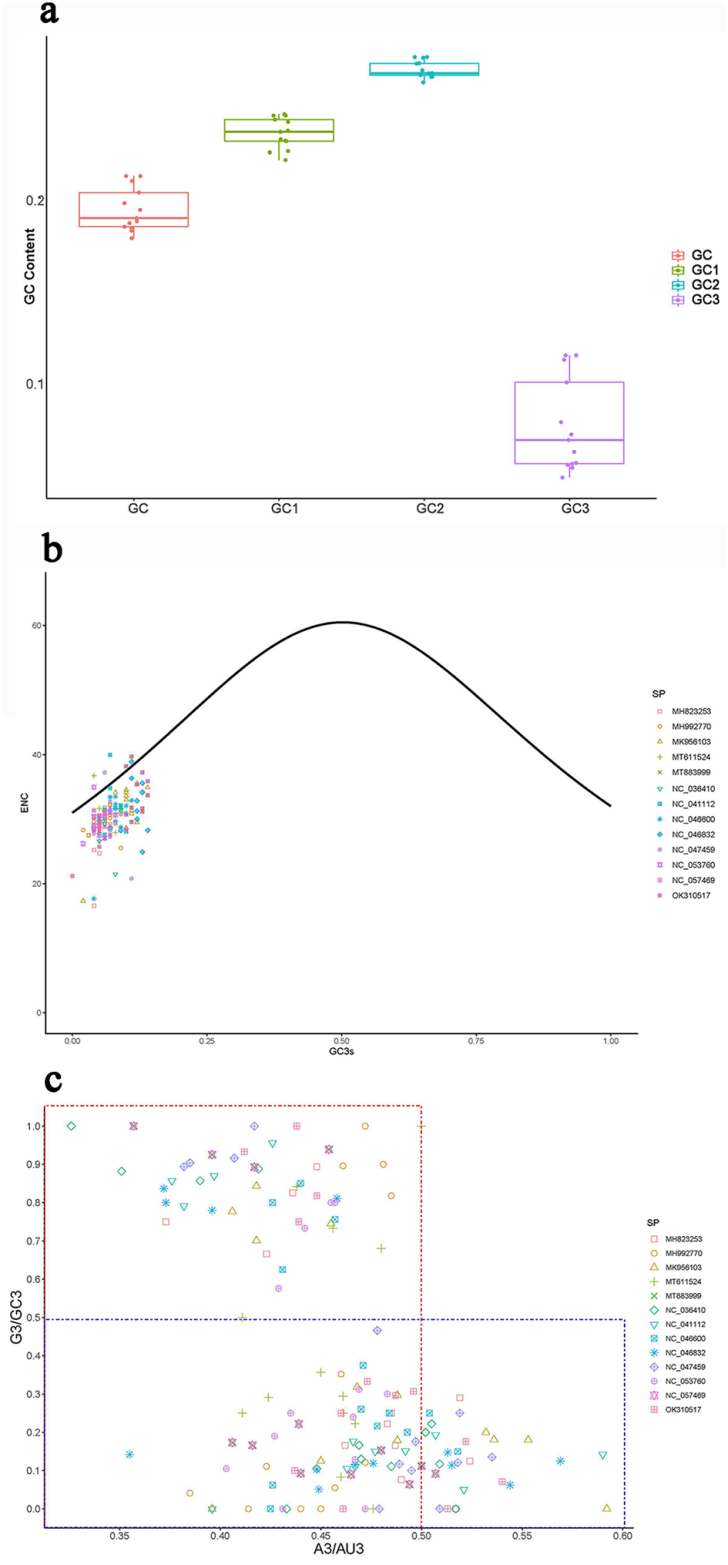
(**a**) The box diagram shows the G + C content at different positions of the condon of PCGs in mitogenomes of 13 Tineoidea species. (**b**) ENC-plot shows the relationship between GC3 values and ENC values for protein-coding genes in mitochondrial genomes of 13 Tineoidea species. (**c**) PR2 bias shows the base content of the third codon of the PCGs of mitogenomes of 13 Tineoidea insects.

In order to further analyze the reasons for the formation of codon bias patterns in mitochondrial genome, we calculated the G + C content of protein coding codons, and analyzed the ENC-plot. The overall G + C content of the mitogenomes ranged from 16.48 to 20.14%, showing that there was little difference in G + C content among the 13 Tineoidea species mitochondrial genomes. The G + C content of different codon positions in the protein coding region are shown in Fig. 3a. The result showed that the G + C content of GC1 and GC2 were higher than that of the overall GC. However, the G + C content of GC3 was lower than that of the overall GC, which was the main reason for the low content of GC in protein coding codons. Among them, GC3 had the largest change in G + C content, which was often used to explain the formation of codon preference.

The ENC-plot analysis revealed that most values were below the expectation curve, and only a few points were above the expectation curve (Fig. 3b). The result indicated that codon preference of protein-coding genes in 13 mitogenomes was mainly determined by natural selection and other factors, while mutation accounted for only part of the reason^29^. In addition, the PR2 bias analysis results indicated that the ratio of A3 to AU3 was 0.326–0.592, and the ratio of G3 to GC3 was 0–1 (Fig. 3c), indicating that the contents of A and U, and the contents of C and G in the third codon were different, showing a certain preference for the use of U and C bases.

**Figure 4:**
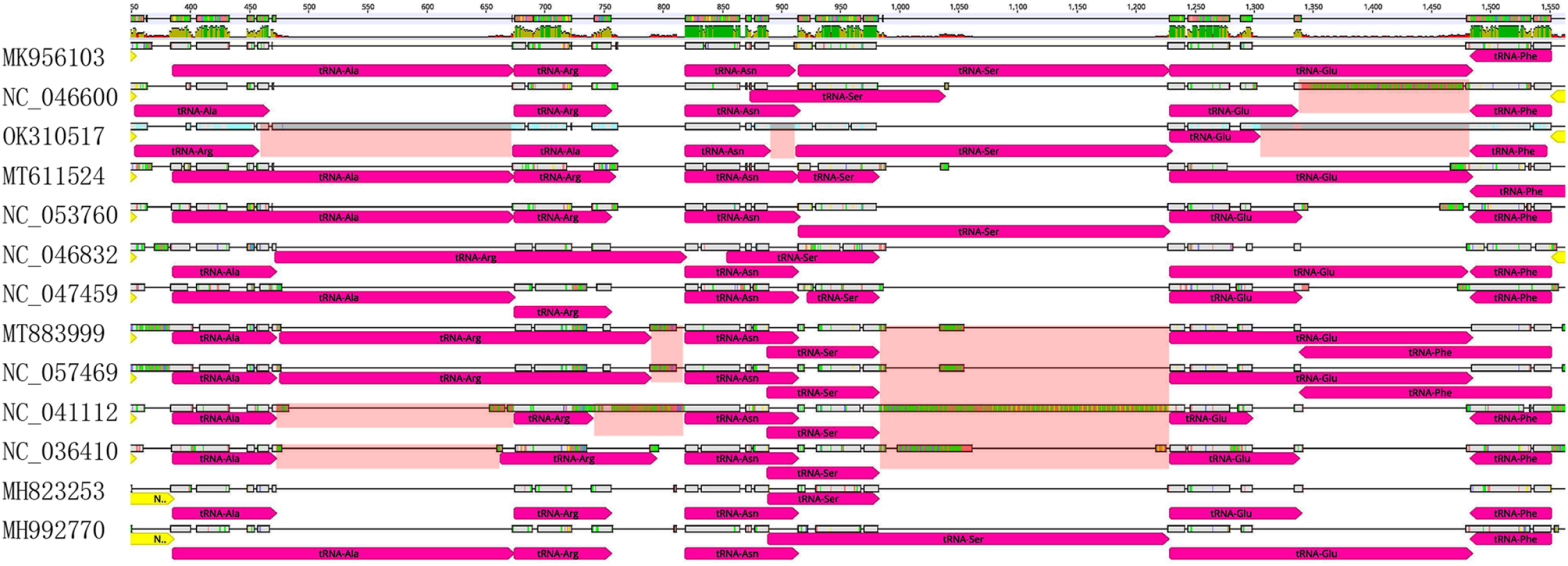
The tRNAs gene arrangement in the mitogenomes of 13 Tineoidea species by Geneious software.

**Figure 5:**
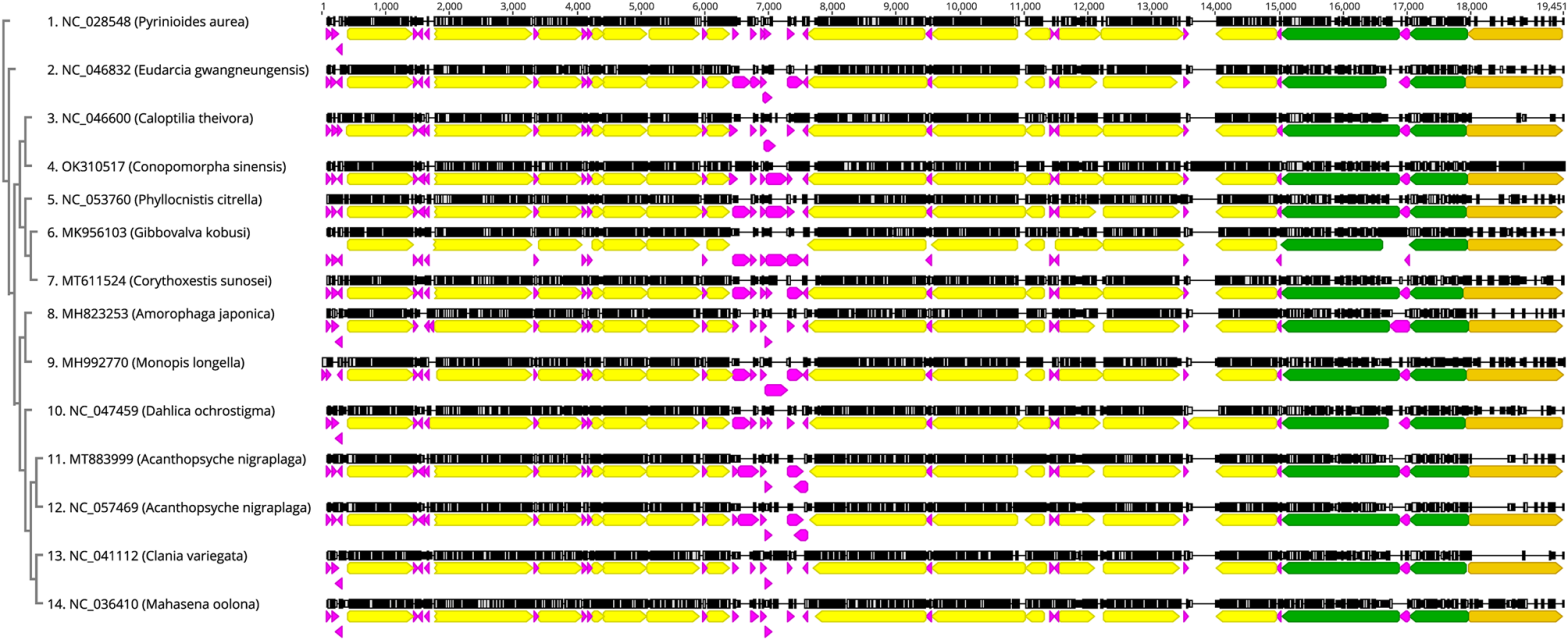
Gene arrangement in mitogenomes of 13 Tineoidea species by Geneious software. The phylogenetic tree is on the left, in which NC_028548 is an outgroup. In the gene rearrangement map, different color regions show different gene regions of mitochondria, yellow: the PCGs; fuchsia: tRNAs; green: rRNAs; and orange: A + T-rich region.

The arrangement of mitochondrial genes is very conserved among insects^10,30,31^. There are larger rearrangements in PCGs and rRNAs genes, and smaller gene rearrangements in tRNAs genes^10^. The mitogenome arrangement was found to be conserved among 13 Tineoidea species (Supplementary Fig. S3 and Fig. 4). However, compared to 12 Tineoidea species, there were two large variation regions in the mitogenome of litchi fruit borer. One of the variation regions was a gene rearrangement in the *trnA*-*trnF* gene cluster of tRNA, with the order of gene cluster *trnA-trnR-trnN-trnS-trnE-trnF* in other Tineoidea species shifting into the *trnR-trnA-trnN-trnS-trnE-trnF*, and insertion of a 205 bp and 178 bp AT repeat sequence between *trnR* and *trnA*, *trnE* and *trnF* in the mitogenomes of *C. sinensis*, respectively (Fig. 4). Another region of variation was between *ND1* and *trnS*, where a 423 bp AT repetitive sequence is inserted in the mitogenomes of *C. sinensis* (Fig. 5).

### Phylogenetic tree analysis

**Figure 6:**
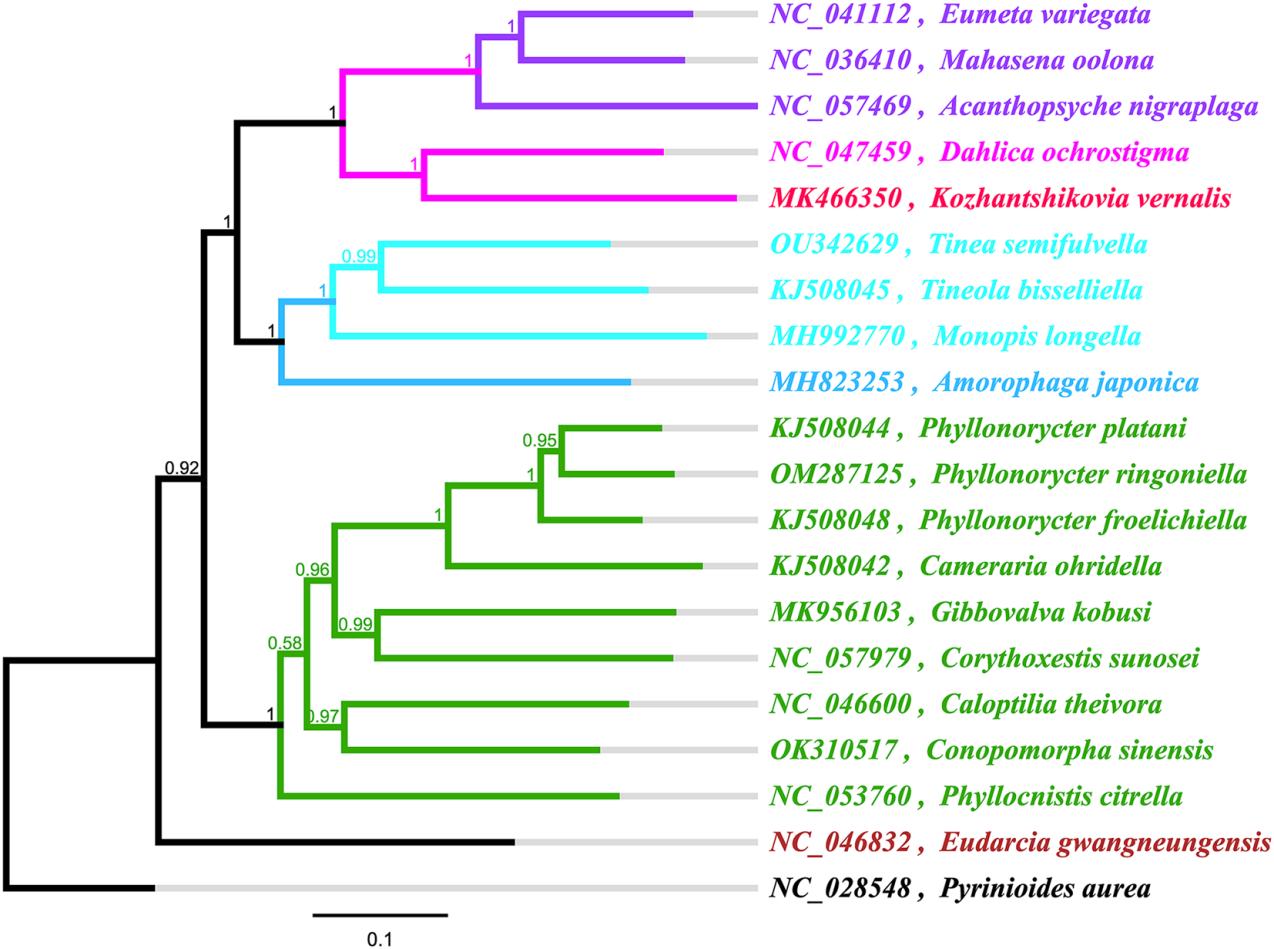
Phylogenetic tree of Tineoidea species based on mitochondrial genome.

The phylogenetic tree constructed based on mitochondrial genome was shown in Fig. 6. Within the Gracillariidae, *C. sinensis* was inferred as a sisiter taxon in a basal position to *Caloptilia theivora*, and *Phyllocnistis citrella* was placed as the most basal lineage in the Gracillariidae. With respect to familial relationships, the Psychidae, Tineidae, Gracillariidae and Meessiidae first form independent branches, and then gather into a large branch and cluster together with the outgroup. The result showed the monophyly of families using mitogenome data, which has also been indicated by morphology-based results^32^.

## Discussion

*Conopomorpha sinensis* Bradley is a pest that causes severe economic damage to litchi and longan. Previous *C. sinensis* research has focused on population life tables, host plants, oviposition selectivity, sex pheromone, pest population prediction, and control technology^2–5^. However, there are few studies on mitochondrial genome and phylogenetic evolution. This study sequenced and analyzed the characteristics of mitochondrial genome of litchi fruit borer. The mitogenome of litchi fruit borer is a typical circular double-stranded structure with a length of 17,050 bp. A total of 37 genes were translated from the mitogenome of *C. sinensis*, and the content of A + T was significantly higher than that of C + G in the PCGs, RNA, control region and the whole mitogenome. These characteristics are consistent with the basic characteristics of insect mitochondrial genome^9,33^.

All of the initiation codons of PCGs were the ATT or ATG codons, except *COI* gene, which uses CGA as the initiation codon. This has frequently been observed in other Lepidoptera^34,35^. All of the PCGs were terminated with TAN or truncated T, which is consistent with insect mitogenomes^10^. The truncated T residue is modified by the post-transcriptional polyadenylation to a complete TAA stop codon^36^. The RSCU values showed that PCGs had obvious codon bias in the mitogenome of litchi fruit bore, which was common in other insects^34,37^. Such as, the codon usage rate of UUU-Phe, AUU-Ile, and UUA-Leu in other insects is also the most frequent^38,39^.

The ENC-plot analysis revealed that codon preference of protein-coding genes in 13 mitogenomes was mainly determined by natural selection and other factors, while mutation accounted for only part of the reason^29^. The main reason affecting the ENC value may be the accumulation of mutation preference of the third codon of the protein-coding gene. In the mitochondrial genome of litchi fruit borer, the high-frequency codons ended with A or U base, which leaded to the increase of codon bias and enhanced the influence of mutation pressure on codon bias. Moreover, based on the base content of the third codon, we performed PR2 bias analysis. Generally speaking, G and C bases, and A and U bases appear in pairs in the third codon, and their contents should be equal. However, PR2 analysis showed that the U content of the third codon was higher than that of A, and the C content was higher than that of G. The results broke the balance of the same G content and C content, and the same A content and U content. The results indicated that the formation of codon bias in insect mitochondrial genome was affected not only by natural selection pressure, but also by mutation pressure. In general, the codon bias of insect mitochondrial genome is affected by many factors, which needs to be confirmed by further research^40–42^.

The 22 typical transport RNA genes were all discovered in the mitogenome of *C. sinensis*. Most of the tRNAs are classic clover-leaf structures, except *trnE* without the pseudoricinr (TΨC) stem and *trnS1* lacking the dihydrouridine (DHU) stem. This abnormal *trnS1* structure is found in the mitogenomes of many other insects, such as *Samia cynthia ricini*^32^, *Cyphonocerus sanguineus klapperichi*^25^, and *Eysarcoris gibbosus*^37^. The abnormal tRNAs are considered to restore and preserve their function during the post-transcriptional RNA editing processes^43^. In addition, some base pair mismatches were found in the tRNAs, including A-A and G-U, and most of the mismatches occurred in the acceptor arm, DHU loop, and anticodon stem. These mismatches also occur in other insects such as *Cyphonocerus sanguineus klapperichi*^25^, Odontiinae^34^, and *Pentatoma rufipes*^44^. Like abnormal tRNAs, mismatched tRNAs can be restored through post-transcriptional mechanisms and thus they do not affect their function.

Similar to that of other Tineoidea species mitochondrial genomes, the gene arragement of the mitochondrial genome of litchi fruit borer is conserved, despite the differences in mitogenome length. Compared with 12 other Tineoidea species, the *trnA*-*trnF* gene cluster of tRNA in the *C. sinensis* mitogenome appears to have a new arrangement pattern. Based on changes of gene position in gene rearrangement, mitochondrial genome rearrangement can be divided into gene shuffling, translocation, and inversion^45^. There are four primary models used to elucidate mitochondrial genome rearrangement, namely, the tandem duplication/random loss (TDRL) model^46^, recombination^47^, the tandem duplication/nonrandom loss (TDNR) model^48^, and the illicit priming of replication by tRNA genes^49^. So far, at least six different mitochondrial gene rearrangements have been demonstrated in Lepidoptera, such as the RNSAEF (the underline indicates a reverse transcriptional direction) rearrangement in *Astrotischeria sp.*^50^ and *Erynnis montanus*^51^; the IMQ rearrangement in *Euripus nyctelius*^52^; and the ARNESF rearrangement in *Mesophleps albilinella*^53^. Due to comparison with the ARNSEF gene arrangement in ancestral insects, the RNSAEF rearrangement only requires inverted transposition, which can be illustrated by the TDRL model^54^. However, the ARNESF rearrangement requires inversion and translocation to achieve this gene arrangement, which can be illustrated by the TDRL model and recombination^53^. According to the results of Geneious analysis, the *C. sinensis* mitochondrial genome has a new rearrangement RANSEF at the ND3 and ND5 junction, which can be best explained by TDRL model^46^. This new arrangement has never been found in other Tineoidea moths or other Lepidoptera. It is unknown whether this new rearrangement pattern is a unique mitochondrial gene rearrangement pattern of the family Gracillariidae or just a unique feature formed during the evolution of *C. sinensis*. Further research is needed to confirm whether this unique arrangement pattern of litchi fruit borer exists in other Gracillariidae or Lepidoptera species.

In addition, a long AT repeated sequence was inserted between *trnR* and *trnA*, *trnE* and *trnF*, *ND1* and *trnS* in the mitochondrial genome of the litchi fruit borer. Although some AT-rich regions were also found in the intergenic regions of mitochondrial genomes of 12 other Tineoidea insects, there were other base sequences in the AT-rich regions, rather than almost completely composed of AT bases like litchi fruit borer. The large number of AT repeats may be an important reason for rRNA rearrangement in the mitogenome of litchi fruit borer. At the same time, due to the existence of these AT repeated sequence, the complete mitochondrial genome of litchi fruit could not be obtained by second-generation sequencing. Combined with the results of the third generation sequencing, the complete mitochondrial genome of litchi fruit borer was obtained. However, the study could not explain the reason for the occurrence of AT repeated sequence, and further research is needed to clarify.

The phylogenetic analysis indicated the family Gracillariidae as a monophyletic taxon with well support. The results showed that Meessiidae was resolved as the most basal lineage of the Tineoidea. Similar phylogenetic results for the familiar relationships was illustrated by Regier et al.^32^. However, some studies have shown the Gracillariidae to be the most basal group for Tineoidea^55,56^. Previous studies have shown that mitochondrial gene rearrangement contains useful information for phylogeny and can be used as a new molecular marker in phylogeny research^57–59^. However, the results of mitogenome analysis of Lepidoptera were inconsistent with this opinion^60^. MIQ and IQM are considered to be the unique arrangement of Ditrysia and non-Ditrysia mitochondrial genome groups in Lepidoptera, respectively. Therefore, mitochondrial genomes of more species are needed to elucidate the phylogenetic relationships of Tineoidea^60^. However, the analysis of tRNA gene rearrangement in Lepidoptera showed that MIQ also appeared in Tischerioidea in the non-Ditrysia group^60^. In addition, a new tRNA gene rearrangement pattern of QIM appeared in Micropterigidae^60^. At the same time, some Lepidoptera insects had unique gene rearrangements, and most of them occured in the region of ARNSEF. *Mesophleps albilinella* in Gelechiidae has the same tRNA rearrangement phenomenon as Zygaenidae, which is ARNESF^60^. In general, the occurrence of gene rearrangement in the mitochgenome is random and has no direct connection with the evolutionary relationship between populations. However, some scholars thought that gene rearrangement may be a common feature of mitogenome in some groups of Lepidoptera^61,62^, which needs further study. In addition, mitochondrial genomes of more species are needed to elucidate the phylogenetic relationships of Tineoidea.

## Conclusion

We sequenced the whole mitogenome of *C. sinensis* by the combination of second-generation sequencing and third-generation sequencing, analyzed the characteristics of its mitochondrial genome by comparative genome, and analyzed the phylogenetic position by constructing phylogenetic tree in the study. The mitogenome of litchi fruit borer is a typical circular double-stranded structure with a length of 17,050 bp. A total of 37 genes were translated from the mitogenome of *C. sinensis*, and the content of A + T was significantly higher than that of C + G in the PCGs, RNA, control region and the whole mitogenome. Compared to the mitogenomes of 12 Tineoidea insects, the mitogenome of *C. sinensis* was the longest, and the control region had the greatest change in size. The content of A + T in the mitochondrial genome was the highest. Further, the gene arrangement of the mitochondrial genome was compared to 12 other Tineoidea species, a 423 bp AT repeat sequence was inserted between *ND1* and *trnS* in the mitogenomes of *C. sinensis*. Meanwhile, the *trnA*-*trnF* gene cluster of tRNA was a new gene rearrangement in the mitogenomes of *C. sinensis*, which needs further exploration. Furthermore, the results of phylogenetic analysis showed that the litchi fruit borer belonged to Gracillariidae, and Gracillariidae was monophyletic. Our findings is helpful to understand the phylogenetic status of litchi fruit borer and the complexity of its mitochondrial genomes. It also provides valuable molecular data for future studies on the genetic structure, differentiation, and evolutionary relationships of *C. sinensis* populations.

## Materials and methods

### Insects rearing

Falling litchi fruits, infested with *C. sinensis*, were harvested from the Institute of Tropic Fruit Trees, Hainan Academy of Agricultural Science. The fruits were placed in a white porcelain plate covered with corrugated paper. *C. sinensis* pupae were collected from the corrugated paper and placed in insect rearing cages until eclosion. *C. sinensis* adults were stored in an ultra-low temperature refrigerator until use.

### DNA extraction and high-throughput sequencing

Genomic DNA of litchi fruit borer was extracted by CTAB method^63^. The quality and quantity of genomic DNA were measured using NanoDrop 2000 (Wilmington, DE, USA). The genomic DNA was qualified for the high-throughput sequencing at the Benagen (Wuhan, China). The second-generation and third-generation sequencing experiments were separately performed according to the standard protocol provided by Illumina and Oxford Nanopore Technologies.

### Mitogenome assembly and analysis

The raw data were filtered to remove low-quality reads and adapters by SOAPnuke (version: 1.3.0)^64^. Illumina sequencing and Oxford sequencing generated 2.1 G of clean data and 6.7 G of pass data, respectively. The clean third-generation data were compared with the local mitochondrial genome database by minimap2^65^, and we treated the matched sequence as the core region of the mitochondrial genome. On this basis, the whole mitogenome was assembled by Unicycler software (version: 0.4.8)^66^, and we corrected the assembled mitogenome using the second-generation data by Pilon software^67^.

The mitogenome structure of *C. sinensis* was annotated using the online software MITOS2, and the annotated information was adjusted by comparing it with Gracillariidae species on NCBI (Table 3). The mitogenomic circular map was produced using OGDRAW web server. The nucleotide skew values were calculated as follows: GC-skew = (G − C)/(G + C), AT-skew = (A − T)/(A + T)^68^. The relative synonymous codon usage (RSCU) values and the effective number of codon (ENC) values of *C. sinensis* mitogenome was computed referring to the method of previous literature^69^.

**Table 3:** The general information for mitogenome of related species in the study.

Family	Species	Size (bp)	Accession number
Gracillariidae	*Gibbovalva kobusi*	15,717	MK956103
Gracillariidae	*Caloptilia theivora*	15,297	NC_046600
Gracillariidae	*Corythoxestis sunosei*	15,511	MT611524
Gracillariidae	*Phyllocnistis citrella*	15,416	NC_053760
Gracillariidae	*Conopomorpha sinensis*	17,050	OK310517
Meessiidae	*Eudarcia gwangneungensis*	15,391	NC_046832
Psychidae	*Dahlica ochrostigma*	15,429	NC_047459
Psychidae	*Acanthopsyche nigraplaga*	15,704	MT883999
Psychidae	*Acanthopsyche nigraplaga*	15,704	NC_057469
Psychidae	*Clania variegata*	15,660	NC_041112
Psychidae	*Mahasena oolona*	16,119	NC_036410
Tineidae	*Amorophaga japonica*	15,027	MH823253
Tineidae	*Monopis longella*	15,541	MH992770

### Comparative mitogenomic analysis

The mitogenomes of related species were queried and downloaded from NCBI (Table 3). The mitochondrial genome of *C. sinensis* was acted as a reference, and the structural variation of mitogenomes between the related species was analyzed by LASTZ (version: 1.04.15)^70^ and Mauve software (version: 2.4.0)^71^. The variation analysis of gene sequencing was compared with the built-in mafft plugin of Genetic Prime (version: 2021.1.1) and then displayed with Genetic Prime^72,73^. The length and base characteristics of mitogenomes were analyzed by statistical analysis. The ENC value and G + C contents of the first (GC1), second (GC2) and third (GC3) codon positions and overall G + C content (GC) of protein-coding region were computed by EMBOSS^74^. We used the base content of the third codon to analyze the parity rule 2 (PR2)^75^. The PR2-bias plot, and ENC-plot were drawn using R pack ggplot2^76^.

### Phylogenetic analysis

The whole mitochondrial genome was used to construct the phylogeny of 19 species from the family of Psychidae, Tineidae, Gracillariidae and Meessiidae. The whole mitogenome alignment was performed by mafft software, and the maximum likelihood (ML) tree was constructed using FastTree software^77^.

### Ethics approval and consent to participate

All experiments conducted in this research were in accordance with the IUCN Policy Statement on Research Involving Species at Risk of Extinction and the Convention on the Trade in Endangered Species of Wild Fauna and Flora. The use of litchi fruits has been licensed.

## Supplementary Information

Supplementary Figure S1.

Supplementary Figure S2.

Supplementary Figure S3.

Supplementary Figure S4.

Supplementary Legends.

Supplementary Table S1.

Supplementary Table S2.

## Data Availability

The Genbank accession number for the whole mitogenome of *C. sinensis* is OK310517.
