# Supplementary figures and images for: Comparative genome and phylogenetic analysis revealed the complex mitochondrial genome and phylogenetic position of Conopomorpha sinensis Bradley
Source: Sci Rep. 2023 Mar 27;13:4989. doi: 10.1038/s41598-023-30570-7 (PMC10042987; doi:10.1038/s41598-023-30570-7)

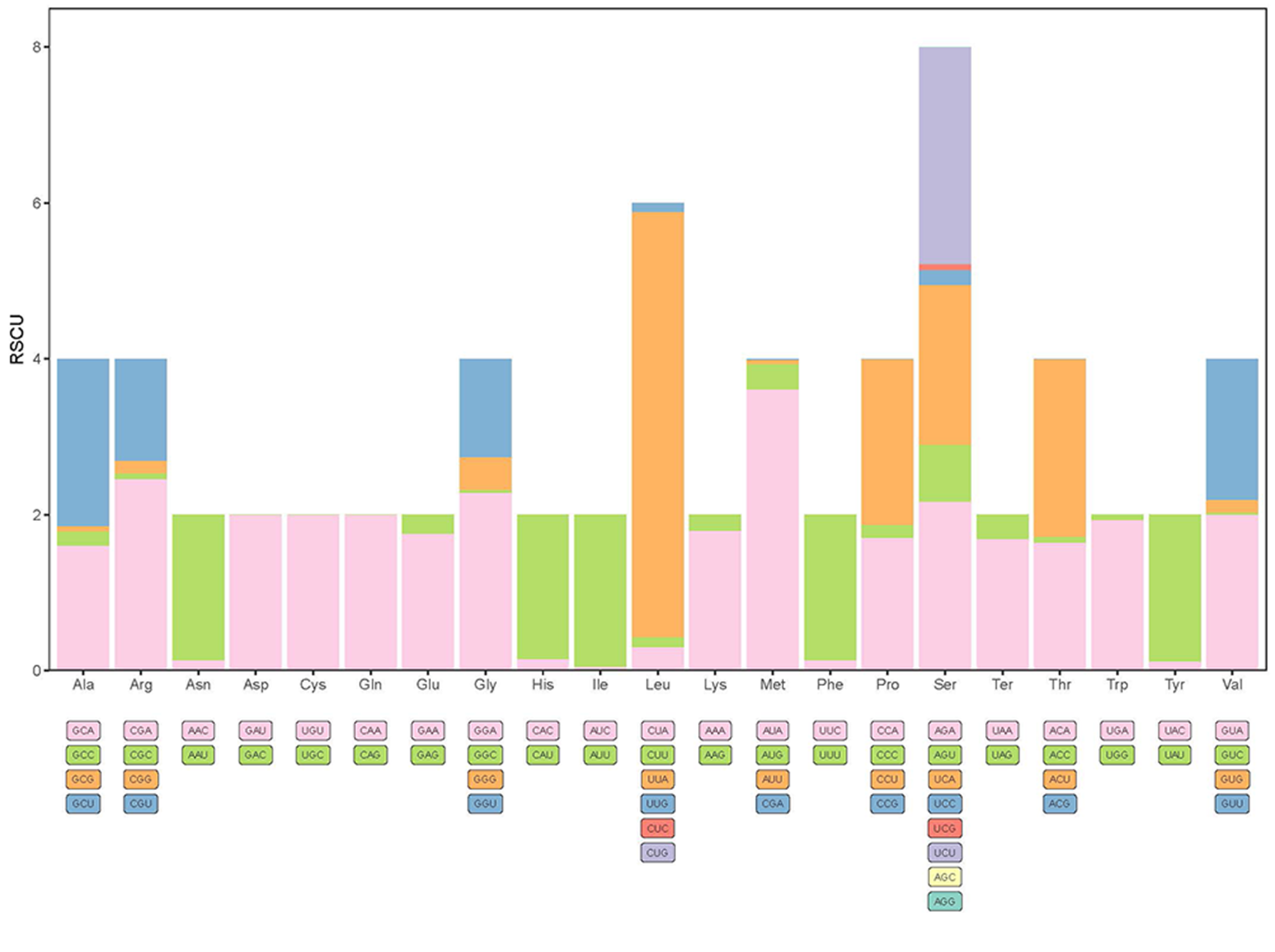

Supplement: Supplementary file 1 — Supplementary Figure S1. [file 41598_2023_30570_MOESM1_ESM.tif]

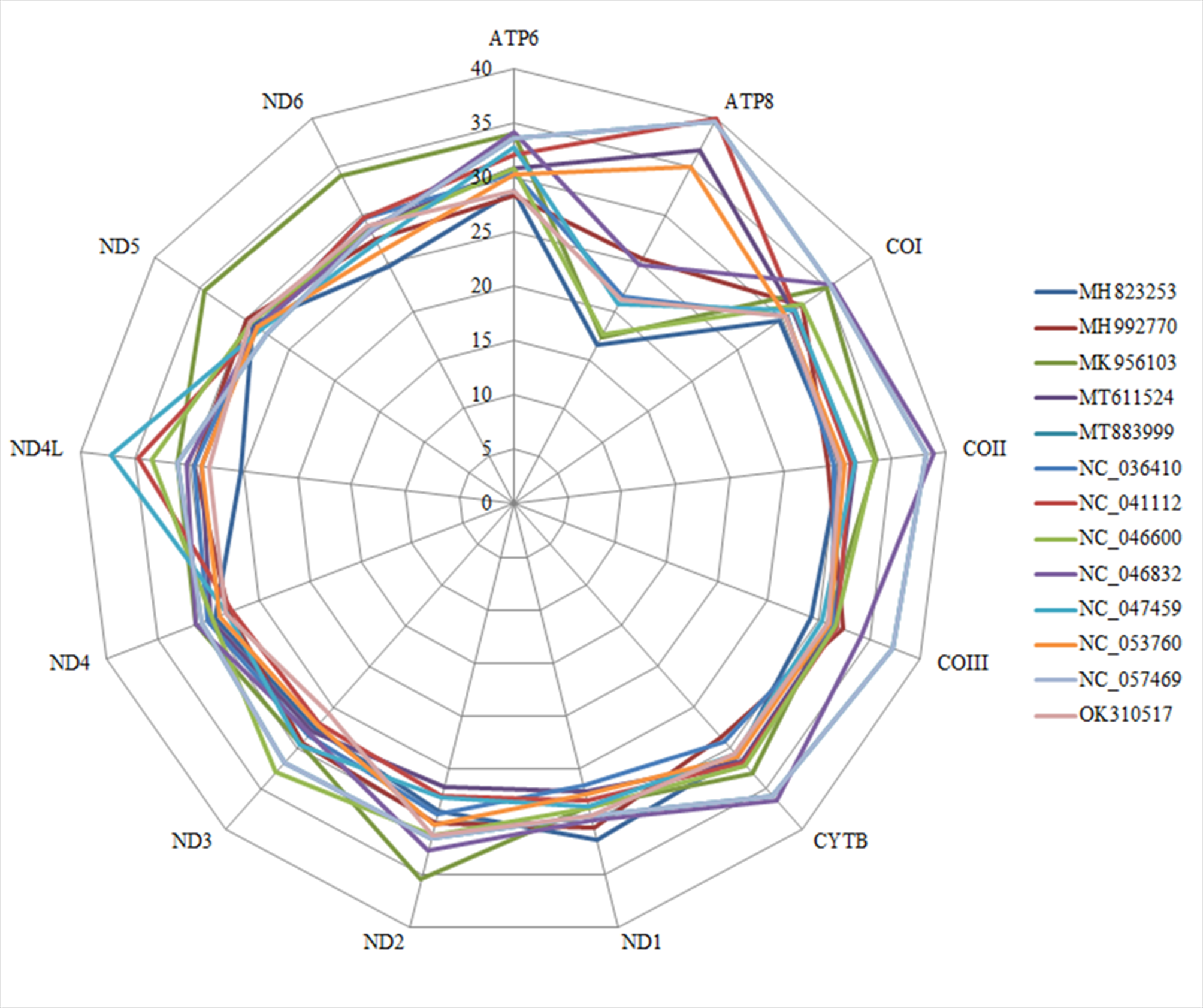

Supplement: Supplementary file 2 — Supplementary Figure S2. [file 41598_2023_30570_MOESM2_ESM.tif]

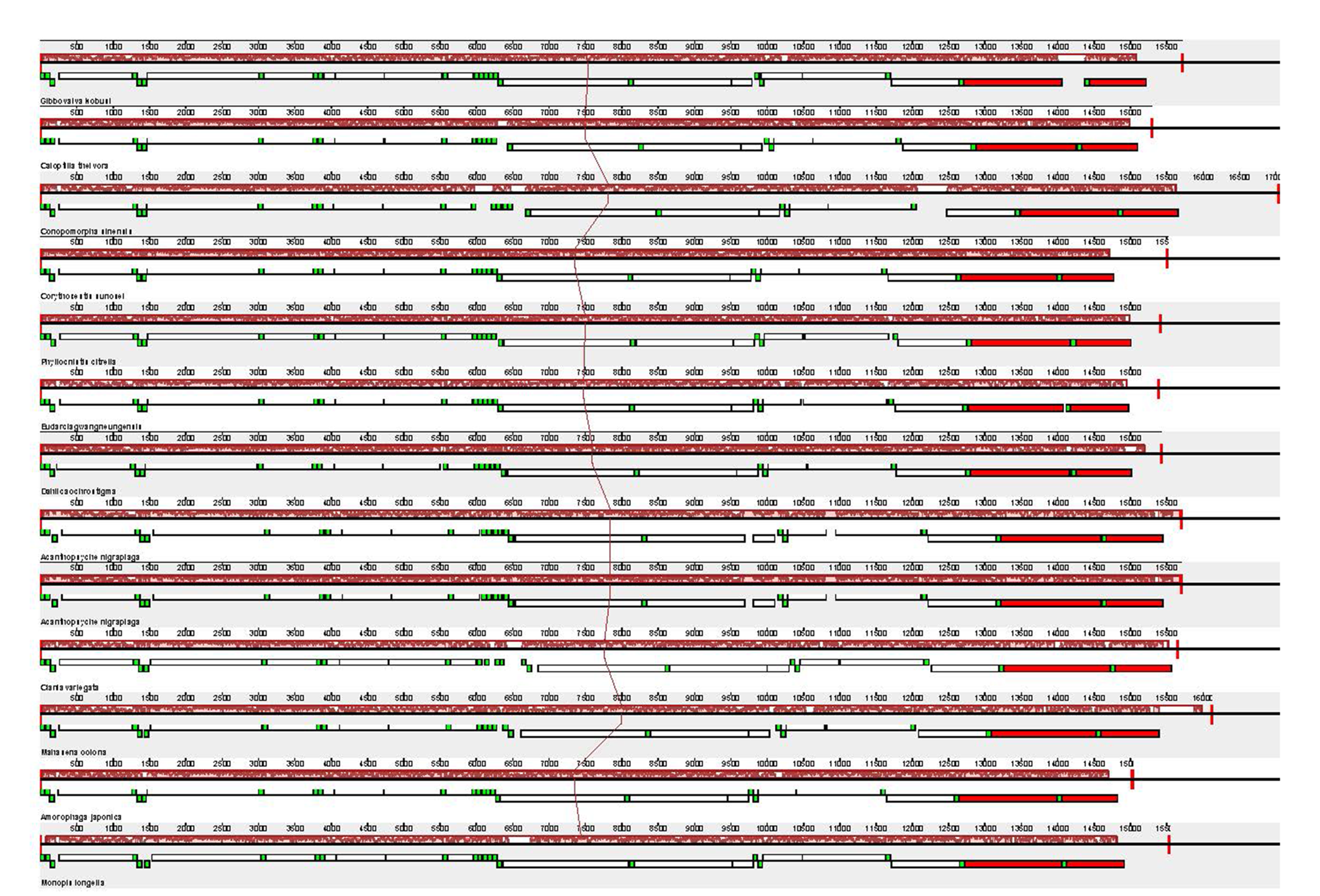

Supplement: Supplementary file 3 — Supplementary Figure S3. [file 41598_2023_30570_MOESM3_ESM.tif]

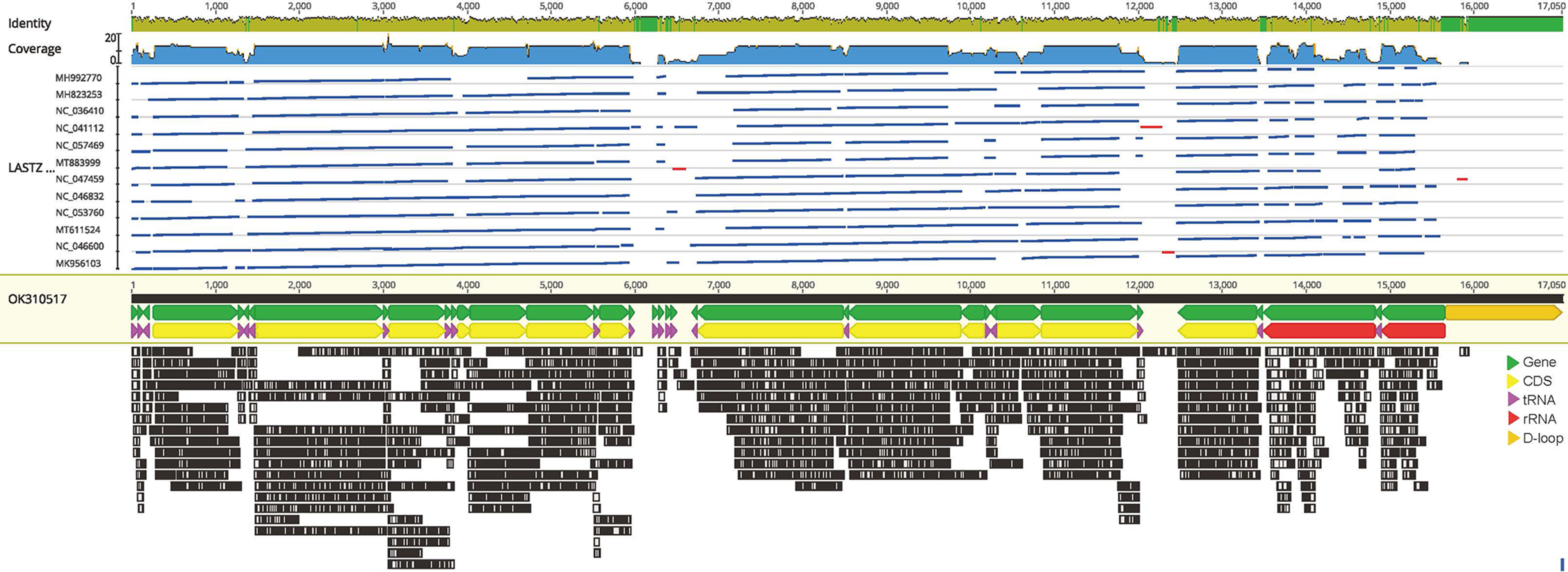

Supplement: Supplementary file 4 — Supplementary Figure S4. [file 41598_2023_30570_MOESM4_ESM.tif]
