# Supplementary material for: Comparative genome and phylogenetic analysis revealed the complex mitochondrial genome and phylogenetic position of Conopomorpha sinensis Bradley
Source: Sci Rep. 2023 Mar 27;13:4989. doi: 10.1038/s41598-023-30570-7 (PMC10042987; doi:10.1038/s41598-023-30570-7)
Supplement: Supplementary file 5 — Supplementary Legends. [file 41598_2023_30570_MOESM5_ESM.docx]

**Figure S1**. Values of RSCU in the PCGs of *C. sinensis*. The horizontal coordinate is codon type, the vertical coordinate is RSCU value, and the bar chart is the cumulative distribution of different codons.

**Figure S2**. The radar diagram shows the ENC values of the PCGs in the mitogenomes of 13 Tineoidea species.

**Figure S3**. Gene arrangement in mitogenomes of 13 Tineoidea species by Mauve software.

**Figure S4**.Gene arrangement in mitogenomes of 13 Tineoidea species by LASTZ3 software.

**Table S1.** Information on 13 Tineoidea species with their complete mitochondrial genome sequences.

**Table S2.** The ENC values of the PCGs in the mitogenomes of 13 Tineoidea species.
